# Proteomics and Its Applications in Cancers 2.0

**DOI:** 10.3390/ijms25084447

**Published:** 2024-04-18

**Authors:** Stanislav Naryzhny

**Affiliations:** Petersburg Nuclear Physics Institute Named by B.P. Konstantinov of National Research Centre “Kurchatov Institute”, 188300 Gatchina, Russia; snaryzhny@mail.ru

Considering the success of our previous Special Issue (SI) “Proteomics and Its Applications in Cancers”, we aimed to attract more publications where cancer proteomics is involved. Altogether, the SI “Proteomics and Its Applications in Cancers 2.0” presents one review and eight research papers. 

Cancer is a systemic malignant degeneration of cells that can affect all human organs. Cancer is among the leading causes of death worldwide (https://www.who.int/news-room/fact-sheets/detail/cancer) (accessed on 11 April 2024) [1]. The main characteristic of cancer is the reorganization of the cell, causing its uncontrolled proliferation. Just one abnormal cell can grow into a tumor, which can penetrate into surrounding tissues and migrate to other organs, i.e., metastasize [2,3]. Since these processes are accompanied by changes in the protein composition (proteome) of the tissue and organism, proteomics can provide detailed information with regard to this. For example, it may reveal key information that can facilitate the search for clinically applicable biomarkers for therapeutic purposes [4]. Proteomics can provide answers to general and specific questions—about the quantity and abundance of proteins, their participation in metabolic pathways, interactions, post-translational modifications (PTMs), as well as synthesis and degradation. Together with other large-scale omics-based studies (genomics, metabolomics, etc.), proteomics provides extensive data which can explain the molecular mechanisms of cancer [5].

The majority of the papers in this SI deals with the study of cancer biomarkers [6,7,8]. This area is an everlasting “hot topic” in cancer studies. It is not surprising that cancer- and biomarker-specific journals publish copious manuscripts devoted to this area. In this regard, the Special Issue of this *IJMS* journal fits the situation very well. The other papers of the SI, not directly involved in cancer biomarkers, offer insight into general aspects of the cellular proteome organization and protein–protein interactions [9,10,11,12].

A specific aspect of cancer biomarkers that is highlighted in this SI that warrants special attention is the topic of immune response, in particular, the omics-based study of epitomics [13]. Epitomics, or protein epitome profiling, is a tool for understanding subprotein-level variation [14]. Recently, studies in the field of proteomics have begun to pay more attention not only to quantitative changes in protein levels, but also to the development of systematic approaches to monitor variation at the level of specific protein forms—proteoforms. These proteoforms can arise due to alternative splicing, PTMs, processing, and degradation [15,16]. Moreover, as a result, these proteoforms that have specific surface structures can expose different epitopes and carry out different functions. These epitopes can be detected using monoclonal antibodies. Accordingly, a robust and analytically validated PEP (protein epitome profiling) technology that detects the variability of epitopes has been developed to characterize the immunogenicity of plasma [14].

To study C9 proteoforms and their relevance to lung cancer, Torny et al. compared the molecular heterogeneity of the C9 epitope in plasma samples from patients with lung cancer and a control group [17,18]. It allowed the authors to find specific C9 proteoforms associated with lung cancer. Accordingly, they identified and classified these specific epitopes of C9. A general scheme of such a proteoform study is shown in Figure 1. 

In the work conducted by Irajizad et al., the authors searched for so-called “cryptoproteins” and found very specific proteoforms that induce autoantibody responses in cancer (particularly in non-small-cell lung cancer) [19]. To this end, proteomic profiles of lung adenocarcinoma (LUAD) cell lines were examined to assess the presence of cryptoproteins. These Ig (immunoglobulin)-bound cryptoproteins were analyzed via mass spectrometry. A total of 420 cryptoproteins were identified in LUAD cell lines, and a database was built for them [19]. 

It should be noted that a comprehensive analysis of the discovery of cancer and biomarkers requires the use of combinations of different approaches and technologies, including proteomics [20]. Here, validation is a critical step, as only independently and reproducibly proven biomarkers will have potential for clinical usage [21]. 

Finally, I would like to thank all contributors, peer reviewers, editors, and the MDPI’s publishing team for their hard work. As a Guest Editor, I hope that this SI will also be of interest for the scientific community.

## Figures and Tables

**Figure 1:**
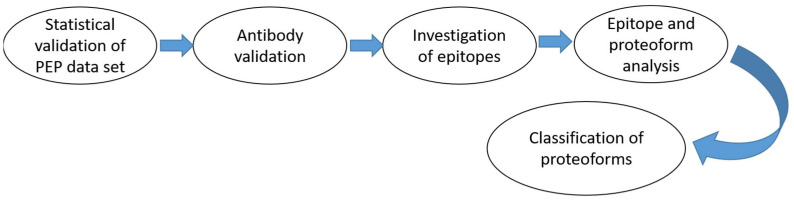
The sequence of experiments in epitope studies.
